# Advancements and Challenges in Addressing Zoonotic Viral Infections with Epidemic and Pandemic Threats

**DOI:** 10.3390/v17030352

**Published:** 2025-02-28

**Authors:** Munazza Fatima, Timothy An, Pil-Gu Park, Kee-Jong Hong

**Affiliations:** 1 Department of Microbiology, Gachon University College of Medicine, Incheon 21936, Republic of Korea; munazzafatima@gachon.ac.kr (M.F.);; 2 Lee Gil Ya Cancer and Diabetes Institute, Gachon University, Incheon 21999, Republic of Korea; 3 Department of Health Sciences and Technology, GAIHST, Gachon University, Incheon 21999, Republic of Korea; 4 Korea mRNA Vaccine Initiative, Gachon University, Seongnam 13120, Republic of Korea

**Keywords:** zoonotic viral infections, emerging infections, viral transmission, pandemic, outbreaks, epidemics, hemorrhagic fever, encephalitis

## Abstract

Zoonotic viruses have significant pandemic potential, as evidenced by the coronavirus pandemic, which underscores that zoonotic infections have historically caused numerous outbreaks and millions of deaths over centuries. Zoonotic viruses induce numerous types of illnesses in their natural hosts. These viruses are transmitted to humans via biological vectors, direct contact with infected animals or their bites, and aerosols. Zoonotic viruses continuously evolve and adapt to human hosts, resulting in devastating consequences. It is very important to understand pathogenesis pathways associated with zoonotic viral infections across various hosts and develop countermeasure strategies accordingly. In this review, we briefly discuss advancements in diagnostics and therapeutics for zoonotic viral infections. It provides insight into recent outbreaks, viral dynamics, licensed vaccines, as well as vaccine candidates progressing to clinical investigations. Despite advancements, challenges persist in combating zoonotic viruses due to immune evasion, unpredicted outbreaks, and the complexity of the immune responses. Most of these viruses lack effective treatments and vaccines, relying entirely on supportive care and preventive measures. Exposure to animal reservoirs, limited vaccine access, and insufficient coverage further pose challenges to preventive efforts. This review highlights the critical need for ongoing interdisciplinary research and collaboration to strengthen preparedness and response strategies against emerging infectious threats.

## 1. Introduction

**Figure 1:**
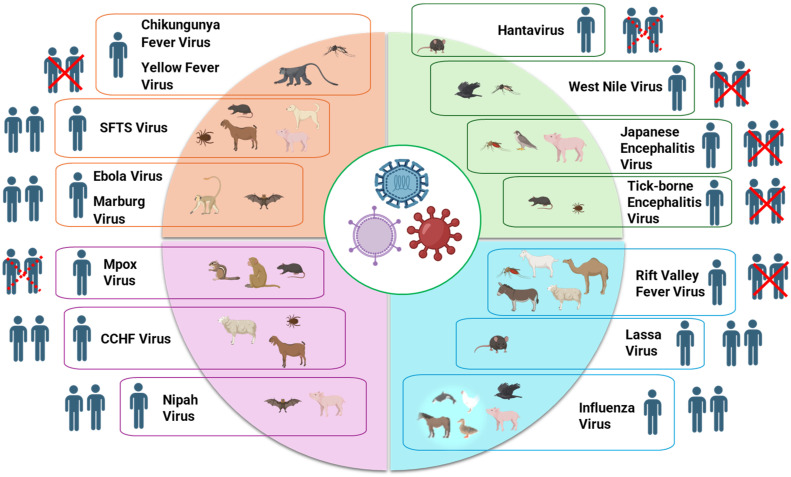
Zoonotic viruses with pandemic potential: Hosts, transmission dynamics, and human contagion risk. Two human images indicate human-to-human transmission, a cross over the image represents no transmission, and a dotted cross indicates rare transmission. The figure is created using BioRender.

Zoonotic viral infections transmitted from animals to humans pose a significant public health challenge due to their ability to cause severe outbreaks and pandemics. Insight into these viruses is essential for developing their effective prevention and control strategies. There is a wide variety of zoonotic viruses, including rodent-borne hantaviruses and arenaviruses, that can seriously infect humans. For example, hemorrhagic fever with renal syndrome can be caused by Puumala hantavirus, usually found in voles [1,2]. Another extremely pathogenic zoonotic virus is the Nipah virus that is usually transmitted from bats to humans via intermediate hosts like pigs. It causes severe respiratory and neurological symptoms and has a high fatality rate [3]. In regions where mosquito vectors are endemic, human health could be at risk from mosquito-borne flaviviruses, including Japanese encephalitis virus and chikungunya virus, which have been detected in wildlife hosts such as pangolins [4]. Numerous zoonotic viruses such as coronaviruses and hantaviruses are harbored in bats and rodents [5]. Viruses harbored by these animals can be transmitted to humans either through direct contact or intermediate hosts. As evidenced by influenza and coronaviruses, intermediate hosts such as pigs and camels play a critical role in the transmission of viruses from wildlife to humans [6,7]. The rising frequency of avian influenza epidemics in poultry that infect humans and the emergence of SARS-CoV-2 emphasize the significance of zoonotic virus infections for global health [8]. These instances highlight the complexity of zoonotic virus transmission and the necessity for comprehensive strategies to lessen their impact on public health. As shown in Table 1, zoonotic viral infections encompass various animal reservoirs and routes of transmission, with the potential for human-to-human transmission, and Figure 1 presents a visual depiction of zoonotic transmission and associated pandemic risks. Table 2 provides an overview of genomic features of zoonotic viruses, as well as existing and emerging interventions to control and prevent their infections.

Studies on the pathogenesis of zoonotic viruses reveal that reservoir hosts, and human mechanisms differ significantly. For instance, rabies and West Nile virus are asymptomatic in their natural hosts, such as bats and rodents, but they cause severe illness in humans. A deeper insight into of these differences might lead to the discovery of important interventions [9]. The emergence of zoonotic viruses is increased by environmental and ecological changes, such as urbanization, deforestation, and climate change, which increase human–animal interactions and promote viral dissemination [8]. Human activities that increase the risk of zoonotic diseases, like live animal markets and pet trade, highlight the need for education and legislation [10]. Robust surveillance and control measures are essential for preventing the transmission of zoonotic viruses. Effective strategies include the monitoring of wildlife and livestock populations, developing vaccines, and implementing biosecurity measures [11,12]. A diversified approach is needed to tackle the challenges posed by zoonotic viral infections. The One Health concept, integrating human, animal, and environmental health, is essential for addressing these complex issues effectively [13].

In this review, we have briefly discussed the research trends and breakthroughs of various zoonotic viral infections, including Chikungunya Fever, Ebola/Marburg Hemorrhagic Fever, Yellow Fever, Monkeypox, Nipah Virus Infection, Lassa Fever, Rift Valley Fever, Tick-borne Encephalitis, Japanese Encephalitis, West Nile Fever, Hantavirus Infection, Crimean-Congo Hemorrhagic Fever (CCHF) and Severe Fever with Thrombocytopenia Syndrome (SFTS). Despite considerable advancements in zoonotic viral research, the variability and evolution of zoonotic viruses continue to pose challenges in developing effective vaccines and therapeutics. The current review aimed to provide a brief overview of the latest ongoing research focused to address zoonotic threats and improve public health.

**Table 1:** Overview of zoonotic viral infections: Reservoirs, modes of transmission, and possibility for human-to-human transmission.

Zoonotic Viral Infection	Causative Agent	Reservoir Host(s)	Transmission Host(s)	Mode of Transmission to Human	Human-to-Human Transmission
Chikungunya Fever	Chikungunya virus	Non-human primates [14,15]	Mosquitoes; *Aedes aegypti* and *Aedes albopictus* [16]	Mosquito bite	No
Ebola/Marburg Hemorrhagic Fever	Ebola virus, Marburg virus	Fruit bats [17,18,19,20]	Gorillas and Chimpanzees [21], African Green Monkey [22]	Contact with body fluids of infected animals	Yes [22]
Yellow Fever	Yellow Fever virus	Non-human primates [23,24,25]	Aedes Mosquitoes [25]	Mosquito bite	No
Monkeypox	Mpox virus	Non-human primates [26,27], rodents [28,29]	Rodents [30], Monkeys [27]	Contact with body fluids or mucosal lesions of infected animals	Rare [30]
Nipah Virus Infection	Nipah virus	Bats (fruit bats) [31], flying-foxes [32]	Bats [33,34], Pigs [35]	Contact with body fluids or respiratory secretions of infected animals, consumption of contaminated date palm sap	Yes [36]
Lassa Fever	Lassa virus	Rodents (multimammate mouse) [37,38]	Rodents [39]	Direct exposure to rodent excreta, bodily fluids or indirect exposure via contaminated surfaces and food	Yes [40]
Rift Valley Fever	Rift Valley Fever virus	Livestock (sheep, cattle, goats, camels, donkeys) [41,42]	Mosquitoes [43,44,45,46]	Mosquito bite, direct contact with body fluids of infected animals	No
Tick-borne Encephalitis	Tick-borne Encephalitis virus	Rodents [47]	Ticks [48,49]	Tick bite	No
Japanese Encephalitis	Japnese Encephalitis virus	Birds [50], pigs [51,52]	Culex Mosquitoes [53,54,55]	Mosquito bite	No
West Nile Fever	West Nile virus	Wild Birds [56]	Culex Mosquitoes [57,58,59]	Mosquito bite	No
Hantavirus Infection	Hantavirus	Rodents; mice [60], rats [61], vole [62] Non-rodents; shrew [63], mole [64]	Rodents [65,66]	Rodent bite or inhalation of aerosolized rodent excreta	Rare (e.g., Andes virus) [67,68,69]
Crimean-Congo Hemorrhagic Fever	Crimean-Congo Hemorrhagic Fever virus	Cattle, goat, sheep, hare, wild boars [70]	Ticks [71,72,73]	Tick bite or direct contact with blood or secretions of infected animal	Yes [74,75]
Severe Fever with Thrombocytopenia Syndrome (SFTS)	SFTS virus	Cats [76], dog [77], cattle [78]; sheep, chicken, minks, pig [79]; goat, rodent [80]	Ticks [81,82,83]	Direct contact with the bodily fluids of an infected animal [84]	Yes [85,86]
Influenza	Influenza A, B, C, D viruses	Wild birds [87]	Horses, Poultry, Duck, Chicken, Turkey, Pigs, Horses, Whales, Seals, Mink [88]	Direct contact with infected animals, aerosols, contaminated surfaces	Yes

**Table 2:** Overview of genomic features, classification, and available countermeasures for zoonotic viruses.

Virus	Genome Type	Genome Structure	Viral Family	Genus	Therapeutics	Licensed/Emerging Vaccines
Chikungunya virus	RNA	ss (+)	Togaviridae	Alphavirus	No standard treatment, NSAIDs, DMARDs [89], supportive care	VLA1553 [90]
Ebolavirus, Marburgvirus	RNA	ss (−)	Filoviridae	Ebolavirus Marburgvirus	mAb114 [91], REGN-EB3 [92], supportive care	rVSV-ZEBOV (Ervebo) [93], no licensed vaccine for Marburg
Yellow fever virus	RNA	ss (+)	Flaviviridae	Flavivirus	Supportive care, analgesics and antipyretics, avoidance of Aspirin and NSAIDs	YF-17D [94]
Monkeypox virus	DNA	ds	Poxviridae	Orthopoxvirus	Tecovirimat (TPOXX or ST-246) [95]	JYNNEOS (MVA-BN), LC16m18 and ACAM2000 [96]
Nipah virus	RNA	ss (−)	Paramyxoviridae	Henipavirus	Ribavirin [97], m102.4 [98], hu1F5 [99]	No licensed vaccine, mRNA 1215 (NCT05398796), ChAdOx1 NipahB ISRCTN87634044
Lassa virus	RNA	ss (−) segmented	Arenaviridae	Mammarenavirus	Supportive care	No licensed vaccine, MV-LASV, EBS-LASV, INO-4500 and rVSVΔG-LASV-GPC [100]
Rift Valley fever virus	RNA	ss (−) segmented	Phenuiviridae	Phlebovirus	Supportive care	No licensed vaccine, TSI-GSD-200, hRVFV-4s and ChAdOx1 RVF [101]
Tick-borne encephalitis virus	RNA	ss (+)	Flaviviridae	Flavivirus	Supportive care	FSME-IMMUN (TicoVac) [102]
Japanese encephalitis virus	RNA	ss (+)	Flaviviridae	Flavivirus	Supportive care	SA14-14-2, IXIARO, ChimeriVax (IMOJEV) [103]
West Nile virus	RNA	ss (+)	Flaviviridae	Flavivirus	Supportive care	No licensed vaccine, ChimeriVax-WN02 [104], HydroVax-001 [105], WN/DEN4delta30 [106]
Hantaviruses	RNA	ss (−) segmented	Hantaviridae	Orthohantavirus	Supportive care	Hantavax licensed in China and South Korea [107]
Crimean-Congo hemorrhagic fever virus	RNA	ss (−) segmented	Nairoviridae	Orthonairovirus	Supportive care	No Licensed vaccine, ChAdOx2 CCHF (ISRCTN12351734)
SFTS virus	RNA	ss (−) segmented	Phenuiviridae	Banyangvirus	Supportive care, favipiravir [108]	No licensed vaccine
Influenza	RNA	ss (−) segmented	Orthomyxoviridae	Alphainfluenzavirus (A), Betainfluenzavirus (B), Gammainfluenzavirus (C), Deltainfluenzavirus (D)	Oseltamivir, Zanamivir, Peramivir, Baloxavir marboxil [109]	Inactivated (Fluzone, Fluarix, FluLaval, Afluria, Vaxigrip), live attenuated (FluMist), recombinant (Flublock) [110,111]

## 2. Chikungunya Virus (CHIKV)

CHIKV is an alphavirus predominantly transmitted by mosquitoes, resulting in significant outbreaks globally, particularly in tropical and subtropical regions. The virus reemerged in 2004, contributing to public health challenges due to its unpredictable outbreak patterns [112]. The disease is characterized by acute symptoms, including fever and severe joint pain, with arthralgia affecting up to 90% of the affected individuals. These symptoms can persist for months or even years, severely impacting their quality of life [113]. Severe cases experienced neurological complications and chronic arthralgia, highlighting the urgent need for effective treatments and prevention strategies [114]. Currently, there are no specific antiviral treatments for CHIKV. However, a recently reported study demonstrated that equine polyclonal antibodies have shown promise in preclinical studies by preventing acute infection in mouse models, suggesting their potential for therapeutic use in human [115]. In the absence of the targeted therapies, supportive care remains the primary method for managing complications. Advancements in diagnostics tools, including extremely sensitive and specific ELISA assays, are crucial for accurate diagnosis and epidemiological studies, which are essential for outbreak management and surveillance [116].

While vaccine development for CHIKV is advancing, the live-attenuated CHIKV vaccine VLA1553 is the first and only licensed vaccine that is currently available [90]. Several clinical trials are in various stages to evaluate the safety and efficacy of VLA1553 in adults, adolescents, and children [117,118,119]. VLA1553 has demonstrated complete protection in nonhuman primates, inhibiting viremia and associated symptoms. This study established a crucial serological surrogate of protection, a critical milestone for vaccine development [120]. Other vaccine approaches, including viral vectors and mRNA vaccines, are also being explored [121]. A virus-like particle (VLP) vaccine has shown safety and a robust immune response in Phase 2 clinical trials [122]. The Phase 1 trial of mRNA-1388 has demonstrated long-lasting humoral responses in adults [123]. The unpredictable nature of CHIKV outbreaks presents challenges for executing vaccine efficacy trials. Understanding the epidemiology of the virus and long-term immunity is vital for formulating efficient vaccine development and deployment strategies [112]. In regions such as India, where CHIKV has caused significant outbreaks, research continues to focus on developing effective treatments and preventive measures, reflecting a global dedication to tackling this medical challenge [124].

## 3. Ebola and Marburg Virus

Various studies highlighted the critical role of zoonotic spillover in the epidemiology of filoviruses, including Ebola and Marburg viruses. A study among bushmeat hunters in Guinea revealed the serological evidence of exposure to filoviruses, indicating that closely related viruses may have circulated before known outbreaks caused by EBOV. This discovery highlights the necessity of incorporating serological, genomic and ecological surveillance for the early detection of possible spillover incidents [125]. Egyptian Rousettus bats have been identified as potential reservoirs for the Marburg virus, with findings from Guinea supporting the hypothesis of local spillover events and serving as a source of human infections [126]. Research has revealed that bats exhibit a muted inflammatory response to MARV and EBOV, with macrophages in the M2 state and altered blood-related physiological system, enabling them to harbor viruses asymptomatically. Understanding these mechanisms could provide insights into developing new therapeutic strategies to effectively treat disease caused by these viruses in humans [127]. In therapeutic intervention, REGN-EB3, composed of Atoltivimab, Maftivimab, and Odesivimab, is the first FDA-approved therapy for Zaire ebolavirus infection. Similarly, Ansuvimab-zykl (mAb114, Ebanga™), another FDA-approved monoclonal antibody directed against the Ebola glycoprotein, has demonstrated efficacy in post-exposure treatment, highlighting the growing potential of monoclonal antibodies in addressing viral infections [91,92].

The recombinant vesicular stomatitis virus (rVSV)-based vaccine, Ervebo, has shown high efficacy in preventing Ebola virus disease (EVD) and is currently licensed for use [128,129]. Several promising vaccine candidates for the Marburg virus are being developed on similar technology. The rVSV-N4CT1-MARV-GP vaccine exhibited complete protection in nonhuman primates when delivered one week before viral exposure [130]. Another candidate, PHV01, conferred complete protection in guinea pigs with a single dose, suggesting its potential for further clinical advancement [131]. Innovative multi-pathogen vaccines against Ebola, Sudan, Marburg, and Lassa viruses have recently been developed utilizing adenovirus (ChAdOx1) and Modified Vaccinia virus Ankara (MVA) platforms. These vaccines have demonstrated robust cellular and humoral immunity and provided protection in animal studies [132]. These advancements suggest a broader strategy to prepare for outbreaks of multiple viral threats.

The Marburg Virus Vaccine Consortium (MARVAC) was formed to expedite vaccine development in response to outbreaks in Guinea and Ghana, addressing various challenges in vaccine development [133]. Furthermore, organizations like the Biomedical Advanced Research and Development Authority (BARDA) is actively involved in advancing vaccine candidates by expediting manufacturing and clinical trial preparations to enhance outbreak preparedness [134]. The persistent threat of filoviruses is evident from recent outbreaks. Recently, Ebola outbreak has been reported in Uganda caused by the Sudan virus. It is the world’s ninth outbreak caused by the Sudan strain. It has resulted in nine cases and one death, indicating a case-fatality rate of 11% [135]. Furthermore, the Congo is also currently experiencing a suspected outbreak of Ebola, for which investigation is ongoing to identify the strain. It has resulted in 12 suspected cases, including 8 deaths [136]. An outbreak of Marburg virus was reported in Rwanda in September 2024. It has resulted in 66 confirmed cases and 15 casualties, and the case fatality rate was 22.7%. The outbreak was declared over in December 2024 [137]. Currently, Tanzania is experiencing a Marburg virus outbreak, confirming 10 cases. Among them, two were confirmed, and eight were suspected. It has resulted in 10 deaths, revealing a case fatality rate of 100%, which is alarming [138,139]. The unpredictable nature of filovirus outbreaks complicates clinical trial design and vaccine efficacy evaluation. To address these challenges, innovative approaches, such as clinical immunobridging with existing vaccines as a benchmark, are being considered to overcome these hurdles [140]. Moreover, tackling logistical difficulties related to vaccine manufacturing, distribution, and post-authorization monitoring is crucial for ensuring real-world effectiveness [141].

## 4. Yellow Fever Virus (YFV)

YFV remains endemic in areas such as Brazil, particularly in the state of Minas Gerais. Non-human primates, which serve as reservoirs, and the environmental conditions like warm/rainy seasons that promote mosquito growth play important roles in virus persistence [142]. The *Sabethes* mosquitoes, especially *Sa. chloropterus* have been recognized as the main vector for YFV transmission in the Cerrado region of Brazil, even in the dry season [143]. Genetic studies indicate that YFV transmission in the state of Rio de Janeiro is associated with multiple introductions and distinct genetic clades, emphasizing the importance of genomic surveillance to track YFV transmission dynamics [144]. Experimental models such as squirrel monkeys are useful for investigating YFV pathogenesis because they show symptoms and immune responses like those found in humans [145]. Studies of the Brazilian strain have identified amino acid polymorphisms in the viral methyltransferase, potentially helping the virus to evade immune detection by altering interactions with host responses, particularly type I interferons [146]. Promising therapeutic compounds are being developed in antiviral research. For example, AT-752 has shown significant effectiveness in reducing viremia and enhancing survival in preclinical models [147]. Sofosbuvir effectively inhibited YFV replication and protected infected mice from death [148]. The isolation of potent neutralizing monoclonal antibodies against YFV envelope proteins provides another promising therapeutic approach, as shown by their efficacy in animal models [149].

The live attenuated 17D vaccine is fundamental to the prevention of yellow fever. Two specific formulations YF-Vax^®^ (17D-204) and 17DD-YFV are licensed in different regions, including the United States and Brazil [150]. The administration of a single dose of the 17D vaccine confers life-long immunity, achieving seroprotection rates of 94% in non-endemic regions and marginally reduced rates of 76% in endemic regions, attributed to a higher seroprotection threshold required for effective immunity. However, diminished seroprotection observed among vulnerable populations, such as children and immunocompromised individuals, indicates a potential requirement for booster vaccination in these groups [151]. There have been reports of rare vaccine-associated adverse events, such as cases of neurological disorders, emphasizing the need for vigilance and effective management by healthcare professionals [110]. Attaining a vaccination coverage of at least 80% is necessary in endemic regions for the prevention of outbreaks [151]. Ongoing research is aimed at providing safer alternatives and fulfilling the increasing global demand for YFV vaccine. Several innovative YFV vaccines are currently in preclinical or early clinical stages of development [152]. A recent Phase 1 clinical trial of SII YFV has shown that the vaccine is safe, immunogenic, and capable of attaining high seroconversion rates [153]. Another study highlighted the significance of the co-administration of the YFV vaccine with tick-borne and Japanese encephalitis vaccines. These combinations were found safe and effective, potentially optimizing vaccination programs for travelers to endemic areas [154]. The continued circulation of YFV across various ecological environments, the rising need for vaccination worldwide, and the possibility of vaccine-related adverse events require the surveillance and refinement of vaccination plans.

## 5. Mpox Virus

The recent rise in Mpox virus cases has resulted in substantial advancement in its diagnosis, treatment, and prevention. Among diagnostic innovations, a loop-mediated isothermal amplification (LAMP) method detects Mpox in 30 min by targeting the N4R gene, providing results comparable to conventional qPCR. This highly specific method is applicable in resource-limited settings due to its simplicity [155]. Another innovative diagnostic assay is MPXV-RCC that combines recombinase polymerase amplification with CRISPR/Cas12a technology to differentiate between Mpox virus clades and provide results within one hour [156]. These tools enhance diagnostic accuracy, especially in outbreak sites. Genomic studies have revealed two main clades: clade 1 (with subclades Ia and Ib), more virulent and endemic to Central Africa, and clade 2 (with subclade IIa and IIb), less virulent and prevalent in West Africa. The global outbreak of clade IIb began in 2022 and continues as of August 2024. During this period, Mpox virus has been reported in approximately 120 countries, with over 100,000 confirmed cases and almost 220 deaths [157]. Superspreading events, international travel, and public gatherings have been identified as crucial drivers of the pandemic. The virus exhibited considerable genetic variation compared to previous strains, indicating an accelerated rate of evolution [158]. This highlights the necessity of robust genomic surveillance and epidemiological studies for tracking viral evolution and transmission [159,160,161]. Recent therapeutic advancement include Tecovirimat, an FDA-approved antiviral for orthopoxvirus infections, demonstrated to be effective in non-human primates and utilized off-label in the US and Canada, with development including a variety of formulations for wider use [162]. Computational screenings of FDA-approved drugs have identified multiple candidates with potential efficacy, though further in vitro and in vivo studies are necessary [163]. Recent studies indicate that drugs such as Naldemedine and Saquinavir have potential triple-targeting effects by simultaneously inhibiting Mpox proteins topoisomerase1, p37, and thymidylate kinase, suggesting further investigation to validate these findings [164].

Considerable progress has been made in vaccine development. During the 2022 global outbreak of Mpox, the non-replicating, Modified Vaccinia Ankara virus based MVA-Bavarian Nordic (MVA-BN) vaccine, a third-generation smallpox vaccine utilized for pre- and post-exposure Mpox prophylaxis, proved effectiveness, with no adverse effects [165]. To enhance vaccine coverage, the FDA granted an Emergency Use Authorization (EUA) permitting intradermal administration, which uses reduced dosages [166]. The worldwide spread of Mpox, particularly in non-endemic areas, revealed the declining smallpox vaccination coverage affecting population immunity. Studies indicate that smallpox vaccines, may offer around 88.8% protection against Mpox. However, there are concerns about the efficacy due to amino acid changes in Mpox strains compared to smallpox vaccine strains [167]. Other approved vaccines include ACAM2000 and LC16m8, derived from Vaccinia virus [96]. The mRNA-based multivalent Mpox vaccine BNT166a showed efficacy in pre-clinical studies [168] and is currently undergoing Phase I/II trials. Additional research is required to fully understand the long-term efficacy and safety of available vaccines against evolving Mpox strains [169,170]. Public health initiatives are being intensified to improve awareness, diagnostic capabilities, and vaccination strategies to more effectively manage the outbreaks.

## 6. Nipah Virus (NiV)

NiV poses a serious threat to public health due to its zoonotic nature and high mortality rate. Recent studies have provided deeper insight into its genomic characteristics and pathogenic mechanisms, which are vital for the development of diagnostics and vaccines efficiently. The disruption of autocrine interferon (IFN) signaling is the fundamental pathway underlying NiV pathogenicity. It triggers by sequestering the signal transducer and activator of transcription (STAT) 1 and 2 proteins in inclusion bodies, blocking their activation, a crucial step in expressing the antiviral gene [171]. Recently developed molecular tests are highly sensitive and suitable for resource-limited settings. These innovations allow rapid responses, essential for combating the epidemic [172]. Moreover, a promising alternative is a novel DNAzyme-based fluorescent biosensor for the detection of NiV RNA, which works on the principles of measuring the fluorescence signal of target RNA, enabling it as a reliable diagnostic assay for surveillance and diagnosis [173]. Beyond diagnosis, significant progress has been observed in therapeutic developments. Among these are potent cross-neutralizing antibodies, such as 1E5, derived from Macaca monkeys, which have demonstrated the ability to neutralize henipaviruses, including NiV. This antibody targets the G glycoprotein of the virus, effectively blocking its entry into host cells. Animal studies have demonstrated the protective efficacy of 1E5, emphasizing its potential as a therapeutic agent [174]. Similarly, human neutralizing antibodies including NiV41 and its mature form, 41-6, exhibited protective efficacy against both NiV and Hendra in animal studies [175]. Another monoclonal antibody, m102.4, targeting the G glycoprotein, is progressing in clinical trials, with Phase 1 clinical trials showing its safety and tolerability [98]. The recently reported hu1F5, targeting the prefusion form of F glycoprotein, has shown higher protection in animal models compared to m102.4, suggesting further development of monoclonal antibodies [99].

In terms of vaccine development, significant progress has been achieved, particularly with candidates such as the recombinant Vesicular Stomatitis Virus (rVSV) vaccine that expresses the NiV glycoprotein. The vaccine demonstrated complete protection against NiV in non-human primates, induced a strong humoral response, and conferred long lasting immunity, making it a strong candidate for further development [176]. Another promising vaccine, PHV02, is a live attenuated rVSV vector vaccine that has shown encouraging results in preclinical trials. In experiments conducted with African green monkeys, it rapidly demonstrated protection against NiV by inducing neutralizing antibodies, even at low levels, which are essential for survival [177]. Despite these advances, only two NiV vaccine trials have been initiated, the first utilizing an mRNA vaccine (NCT05398796) and the second utilizing the recombinant adenovirus candidate vaccine (ISRCTN87634044) [178]. Although significant progress has been achieved, challenges remain in developing effective countermeasures against NiV. Considering that NiV has the potential to cause widespread outbreaks, like the COVID-19 pandemic, it is essential to prioritize preparedness and rapid response strategies. The WHO has emphasized the importance of ongoing investigation into diagnostics, treatments, and vaccines, while establishing strategic milestones for the next six years to improve rapid response capabilities for future outbreaks [179,180].

## 7. Lassa Virus (LASV)

LASV, the cause of Lassa Fever (LF), poses a significant threat to public health because of its high morbidity and mortality. Currently, there is no approved vaccine or treatment for human use, prompting research to focus on improving diagnostic tools, exploring animal models, and progressing vaccine and therapeutic studies. The genetic diversity of LASV presents obstacles in developing reliable diagnostic tools. However, evaluations of RT-PCR assays and commercial kits have demonstrated that the GPC RT-PCR/2007 assay and the Mabsky kit have excellent sensitivity and specificity for detecting multiple LASV strains, making them suitable for a wide range of diagnostic applications [181]. In addition to the diagnostic advancements, significant progress has also been achieved in developing animal models. A novel guinea pig model has been developed using the Nigerian LASV clade III strain. This model effectively replicates vital disease characteristics, including high fever and organ-specific viral loads, providing a reliable choice for testing vaccines and drugs in preclinical studies [182].

Promising advancements have been made in LASV vaccine development, and four candidates including MV-LASV, EBS-LASV, INO-4500 and rVSVΔG-LASV-GPC have progressed to clinical trials [100]. Additionally, inactivated rabies-based Lassa vaccine LASSARAB is likely advancing to the Phase 1 trial. It has demonstrated humoral immunity, effectively protecting nonhuman primates from infection [183]. Another vaccine that contains recombinant measles virus expressing the LASV nucleoprotein and glycoprotein is referred to as MeV-NP. It provided rapid protection in cynomolgus monkeys following a single injection, even when administered shortly prior to LASV exposure. This vaccine shows promise in addressing outbreaks, though it is not effective as a post-exposure treatment [184]. Studies of immune responses in Lassa Fever survivors have identified potential vaccine targets, particularly certain regions of the LASV glycoprotein and nucleoprotein, which could guide the development of more effective vaccines [185]. On the therapeutic side, fragment-based drug discovery has identified two compounds, F1920 and F1965, as potential inhibitors of LASV entry into host cells. These compounds inhibit the fusion of virus and host cell membranes, laying the basis for the development of antiviral drugs [186]. In addition, the nucleoprotein of LASV has been identified as a promising therapeutic target because of its critical role in the viral life cycle, and inhibitors targeting this protein can interfere with viral replication [187]. The continued investigation of LASV pathophysiology and the molecular mechanism of virus host interactions are essential for the advancement of effective vaccines and therapeutics. Implementing public health measures to reduce rodent exposure and improve housing situations in endemic regions is essential for controlling Lassa Fever transmission.

## 8. Rift Valley Fever Virus

Rift Valley Fever (RVF) is a zoonotic disease posing a significant risk to public health, particularly in endemic regions. Recent studies have provided valuable insights for improving preventive and control measures. RVF virus infection leads to significant host–pathogen interactions, particularly within in liver and spleen tissues. Transcriptome analysis revealed that the virus activates interferon-mediated pathway resulting in hepatocyte necrosis and the downregulation of metabolic enzymes essential for maintaining homeostasis. The protein LRP1 has been identified as a potential factor determining RVF virus tissue tropism, enhancing our understanding of its pathogenesis in natural hosts [188]. RVF virus employs non-structural (NS) proteins to evade the host’s immune response, disrupt the cell replication cycle, and induce cytopathic effects. These proteins inhibit interferon signaling pathways, which is crucial for antiviral defense [189]. In addition, NS proteins are associated with nuclear translocation of active caspase-3, contributing to the formation of inclusion bodies in infected cells [190].

In Uganda, epidemiological surveillance has revealed sporadic outbreaks of Rift Vally fever with a mortality rate of 42%, primarily associated with direct contact with livestock, highlighting the zoonotic nature of the disease [191]. Genomic surveillance using multiplex amplicon PCR technology has been developed to quickly generate sequence data, enabling the rapid characterization and genotyping of viruses during outbreaks [192]. Significant progress has also been made in vaccine development. In the past, formalin inactivated TSI-GSD-200 has been administered to high-risk individuals. However, stricter regulations and inactivation validation hinder further production. It highlights the need for safer alternatives. Promising RVF vaccine candidates including viral vector-based ChAdOx1 RVF and live attenuated hRVFV-4s have progressed to clinical trials [101]. Adenoviral vector vaccines Ad5-GnGcopt expressing the RVF virus glycoprotein has demonstrated robust immune responses and full protection in preclinical mouse models [193]. Another vaccine candidate, Ad4-GnGc, has generated strong neutralizing antibodies and cellular immune responses, providing sterilizing protection against RVF virus in mice [194]. Although live attenuated veterinary vaccines are available, such as Smithburn (SB) and Clone 13 (Cl. 13), their use is restricted due to the residual pathogenicity and potential risk of reversal. These vaccines demonstrated high lethality when administered to mice intranasally, indicating the need for the development of safer and more effective vaccines for human [195]. Despite these promising advancements, the complex epidemiology of Rift Valley Fever virus involving multiple hosts and vectors continues to pose challenges, necessitating further research to improve the safety and efficacy of vaccines for human application.

## 9. Tick-Borne Encephalitis Virus (TBEV)

Recent advancements in TBEV research have improved our understanding of viral epidemiology and refined strategies for its prevention and control. Even with the availability of vaccines, TBE remains a major public health issue in Europe and Asia, as evidenced by the rising number of cases and instances of breakthrough infections. This increase in TBE cases in Europe has been attributed to various factors, such as changes in human behavior, landscape modifications and climate change, emphasizing the need to consider these factors in public health policies [196]. To tackle these challenges, advancements in vaccine development focus on enhancing the efficacy of existing vaccines (FSME-IMMUN^®^ and Encepur^®^), widely administered for TBEV prevention. Genetic variations among TBEV strains and suboptimal vaccine coverage resulted in breakthrough infections. The latest findings have revealed that a combination of FSME-IMMUN^®^ and Encepur^®^, has been useful to improve the effectiveness of vaccines against different strains of TBE virus [197,198]. Beyond these initiatives, research continues to identify novel vaccine candidates in response to the rising prevalence of TBE. For instance, recombinant influenza and MVA vectors expressing TBEV non-structural protein 1 (NS1) [197] and the recombinant MVA vaccine that expresses TBEV pre-membrane (prM) and envelope (E) proteins have shown protection in mice, indicating a promising direction for the development of more effective vaccines [199]. Recent discoveries have also revealed a novel neutralizing epitope on envelope glycoprotein E of TBEV, showing an effective response in mice and reflecting its strength as potential candidate [200]. T cells play a pivotal role in the immune response to TBEV, influencing both protection and progression of the disease. A better understanding of the interactive pathway of T cells with TBEV could be useful to develop potential vaccine candidates, reflecting higher efficacy [201]. A recently identified TBEV-Eu strain in the Netherlands exhibits unique growth patterns compared to previously known strains. It highlights the significance of ongoing surveillance and characterization of TBEV variants to revise vaccine development and public health initiatives effectively [202].

## 10. Japanese Encephalitis Virus (JEV)

The JEV has been able to spread to new regions due to its increased genetic diversity over the past ten years, as demonstrated by the most recent outbreak in Australia. This change highlights the need for enhanced monitoring and preventative measures, such as improved immunization techniques and more robust mosquito control initiatives [54]. Previously limited to Indonesia, the emergence of JEV genotype IV in Australia highlights the virus potential to disseminate to new endemic areas and calls for a “One Health” approach to JEV surveillance in a variety of ecological settings [203]. This emergence has exposed diagnostic challenges, particularly the requirement for enhanced PCR assays to identify new genotypes. For accurate diagnosis and effective surveillance, universal primers that target conserved regions of flaviviruses and modern real-time PCR detection are essential [204]. JEV’s neurotropic nature is especially alarming because they impact the central nervous system, causing acute inflammatory damage and neuronal death. In order to better understand virus-induced peripheral nerve injury, such as demyelination and axonal degeneration, animal models have been developed. These models offer important insights into how the virus affects the functioning of the nervous system [205]. Studies on the molecular mechanisms of JEV infection have revealed the role of cytokines and receptors in disease pathophysiology, offering information for potential treatment strategies. Comprehending these mechanisms is crucial for creating treatments that target the inflammatory reaction induced by JEV infection [206]. Toll-like receptors (TLRs) mediate the inflammatory response to JEV, especially TLR2, which generates a potent inflammatory response in microglia and exacerbates the illness [207]. The lack of available treatments and our incomplete knowledge of the pathophysiology of the JEV highlight the urgent need for additional research [208].

WHO approved vaccines include the inactivated vaccine IXIARO, live attenuated vaccine SA14-14-2, and live recombinant chimeric vaccine ChimeriVax-JE (IMOJEV) [103]. Despite the existence of effective vaccines, Japanese encephalitis remains the most common cause of viral encephalitis in children, primarily in Southeast Asia. A study revealed that a majority of the JEV affected children were from rural areas and have low socioeconomic backgrounds [209]. A greater number of affected children belonged to farmers or farm laborer families [210]. JEV spread through mosquitoes and in rural areas, its control challenging, with stagnant water in the fields promoting breeding and the transmission of mosquitoes [211]. Among children, immunity is weak, and inadequate vaccination coverage is the most common cause of JEV, as evident from a study in which 80.5% of the infected children were found unvaccinated [209,212]. It indicates that mass vaccination programs are required to fully cover target populations. Numerous pediatric survivors of Japanese encephalitis report neurological sequelae and decreased social engagement. Malnutrition, a low Glasgow Coma Scale (GCS), and a lack of vaccination are all important indicators of a poor prognosis, highlighting the significance of early intervention and vaccination in improving outcomes [213]. Beyond its impact on public health, recent studies have investigated JEV applications in cancer treatment. For example, a live attenuated strain of JEV has demonstrated promise as an oncolytic virus for glioblastoma treatment, promoting antitumor immunity and preventing tumor growth. These discoveries broaden the scope of JEV research and provide new strategies for addressing critical oncology issues [214].

## 11. West Nile Virus (WNV)

There are no approved vaccines or treatments for West Nile Fever, but research has revealed promising therapeutic targets and offered key insights into viral epidemiology and immune response. Genomic analyses of WNV epidemics have identified the viral lineage and its ability to overwinter, with evidence indicating that migratory birds facilitate the movement of WNV strains between Europe and Senegal [215,216]. Studies on domestic geese have also highlighted the significance of sentinel animals in the surveillance of WNV, as these birds can exhibit viremia without serving as hosts for significant amplification [217]. Understanding immune dynamics is another critical area of emphasis. Cytokines play a dual role in WNV infection, with certain cytokines contributing to viral clearance, while others cause neuroinflammation and tissue damage. Unraveling these underlying mechanisms are critical for developing treatments that can modulate immune responses and improve patient outcomes [218]. Promising therapeutic approaches encompass single domain antibodies (sdAbs), such as sdAbA10, which block virus host interactions, suggesting potential for alleviating West Nile virus-induced neuropathogenesis [219].

Advancements in vaccine development have shown promise, as several vaccine candidates for WNV are undergoing clinical trials [220]. None have progressed beyond Phase 2, highlighting challenges in demonstrating adequate efficacy and safety for approval [221]. A key obstacle is the immunological cross-reactivity between flaviviruses, which hinders the development of a specific and effective WNV vaccine without adverse effects on immunity to other flaviviruses [222]. Recent studies have shown that employing a mutated fusion loop (FL) in the E protein can reduce cross-reactivity with other flaviviruses, potentially lowering the risk of antibody-dependent enhancement (ADE) while maintaining protection against WNV [223]. Another study has reported a plant-made vaccine candidate utilizing virus-like particles (VLPs) and displaying the WNV E protein domain III, which elicited strong humoral and cellular immunity in mice with reduced ADE risk against related flaviviruses like Zika and Dengue [224]. Adapting strategies for related viruses such as Zika and investigating new vaccine platforms present significant potential for future breakthroughs in the fight against WNV [225,226]. Although these studies offer potential vaccine candidates, the transition from preclinical models to a licensed human vaccine requires comprehensive laboratory validation and clinical trials. The complexity of WNV and its interplay with other flaviviruses poses challenges, but ongoing research continues to advance the field toward effective preventive solutions.

## 12. Hantavirus

Hantavax is a whole virus inactivated vaccine against Hantaan virus (HTNV) or Seoul virus (SEOV), licensed for use in the Republic of Korea and China. However, its protective efficacy remains uncertain, and the immunological correlations of protection are inadequately comprehended. It has shown variable effectiveness, highlighting the need for further research [107]. There is no effective treatment for hantavirus infections, including hantavirus cardiopulmonary syndrome (HCPS) and hemorrhagic fever with renal syndrome (HFRS). Among therapeutic advancements, a significant development is the findings of SNV-42, a potent human monoclonal antibody that neutralizes Sin Nombre virus (SNV), a prevalent hantavirus. This antibody targets the vial envelope and interferes with receptor recognition and fusion during host cell entry, offering a molecular blueprint for human neutralizing antibody responses to hantavirus infection [227]. Another study broadened the repertoire of monoclonal antibodies targeting hantavirus nucleocapsid proteins. These antibodies have shown cross-reactivity with different hantavirus strains, indicating their potential application in a wide range of diagnostics and therapeutics [228].

Studies have focused on DNA vaccines, including a Phase 2a clinical trial assessing a bivalent DNA vaccine targeting HTNV and Puumala virus (PUUV). This vaccine, administered via intramuscular electroporation, elicited neutralizing antibodies in human [229]. Computational analysis that identifies viral motifs within the hantavirus nucleoprotein may guide future vaccine design by revealing potential targets associated with virulence and immune modulation [230]. Databases like HantavirusesDB, which include genomic and proteomic data, are expediting vaccine development and mapping potential therapeutic strategies [231]. The urgency to develop vaccines is particularly apparent in regions with high hantavirus prevalence, such as the Balkans, where the Dobrava serotype is widespread. Research on HFRS in Romanian children highlighted the importance of including hantavirus in the differential diagnosis of acute kidney injury and thrombocytopenia [232]. To understand the immune response to hantavirus, studies have revealed the essential function of CD4^+^CD^8+^ double-positive T cells during Hantaan virus infection. These cells exhibit characteristics like those of cytotoxic T cells, indicating their role in antiviral defense [233]. Furthermore, advancements have been achieved in predicting hantavirus outbreaks via geospatial modeling that delineates habitats and ecological factors that increase the risk of disease transmission from rodent hosts to humans. This approach aims to predict outbreaks to facilitate the execution of more targeted public health measures [234]. The complex interdisciplinary challenge of forecasting Hantavirus diseases, along with the urgent need for efficacious vaccines, continues to be a primary focus for future research.

## 13. Crimean-Congo Hemorrhagic Fever Virus (CCHFV)

CCHFV poses health risks, especially in endemic regions, and the lack of a specific antiviral therapy highlights the need for in-depth understanding of the disease epidemiology, refined diagnostic methods, and the exploration of vaccine candidates. The potential application of CCHFV as a bioterrorism agent necessitates the additional investigation of its biology and epidemiology [235]. CCHFV has been reported in over 30 countries, with notable outbreaks occurring in Africa, Eastern and Western Europe, and Asia [236]. The high seroprevalence of CCHFV antibodies in Ugandan livestock demonstrates the need for a robust surveillance program, essential for identifying environmental hazards and preventing human infections, particularly in regions with high tick prevalence [237]. Significant advances in diagnostics facilitate these efforts. A newly developed reverse-transcription loop-mediated isothermal amplification (RT-LAMP) detection method offers superior sensitivity than RT-qPCR and enables the detection of different CCHF genotypes. This portable, user-friendly method is particularly beneficial in resource-limited settings where CCHF is prevalent [238]. Additionally, a multiplex assay was designed for detecting antibodies against CCHFV nucleocapsid protein and glycoproteins in ruminants. This test is crucial for monitoring the immune response in asymptomatic animal carriers [239].

Innovative strategies are advancing CCHFV vaccine development. A chimpanzee adenoviral vector vaccine (ChAdOx2 CCHF) has demonstrated humoral and cellular immunity, as well as 100% protection in a mouse CCHF model [240]. The first-in-human trial to access its safety is ongoing (ISRCTN12351734). Furthermore, several CCHFV vaccine candidates utilizing various platforms are in preclinical assessment. A baculovirus surface display system for CCHFV has demonstrated potential, with glycoprotein Gn-based vaccines inducing strong humoral and cellular immunity in mice [241]. Subunit vaccines containing nucleoprotein (NP) and glycoprotein components have also shown promise, with NP alone offering full protection in mice and its combination with GP38 further reducing morbidity [242]. Similarly, a replicating RNA vaccine that expresses the CCHFV nucleoprotein and glycoprotein precursor provided significant protection in rhesus macaques, inducing robust non-neutralizing immunity [243]. Additionally, computational methods have been employed to design multi-epitope vaccines targeting CCHFV structural glycoproteins. These vaccines are anticipated to be antigenic and non-allergenic, potentially eliciting roust immune responses, and represent a prospective avenue for future vaccine development [244,245]. Despite these advancements, obstacles persist in achieving a universally effective vaccine due to variable efficacy among platforms and the need for expensive clinical trials. The increasing geographic spread of CCHFV and its tick vectors further highlights the urgency for effective preventive measures [246,247].

## 14. Severe Fever with Thrombocytopenia Syndrome Virus (SFTSV)

SFTS is a tick-borne viral illness with significant public health risks, particularly in East Asia. Recent research has improved our understanding of its pathophysiology, risk factors, and prospective therapies and prevention. SFTS is characterized by a transient hyper proliferation of lambda-type monoclonal plasma cells in the bone marrow, which corresponds with disease severity and signifies compromised antibody-mediated immunity [248]. Smoking has become a significant risk factor, particularly for invasive pulmonary aspergillosis (IPA), with the prognosis of patients with SFTS worsening as exposure intensifies [249]. Immunological research offers deeper insights into SFTS pathogenesis. The NS proteins of SFTSV inhibit the type I interferon response, which is essential for its pathogenicity and ability to escape the host immune system [250]. Research involving SFTSV infected rhesus macaques has revealed significant changes in immune cell populations and cytokine profiles that correspond to mild human symptoms. These findings highlight SFTS induced immune alteration and proposed potential targets for therapeutic intervention [251].

Progress in SFTS vaccine development has yielded promising results in preclinical studies across various platforms. A B-Propiolactone inactivated SFTSV vaccine induced robust humoral and cellular immune responses in mice, offering strong protection against viral challenges. The addition of adjuvants such as Al(OH)3 further enhanced its immunogenicity significantly [252]. An rVSV-based vaccine expressing SFTSV glycoproteins provided full protection in mouse models, with glycoprotein mutations improving stability and assembly, indicating potential for further development [253]. Heterologous vaccination approaches, comprising priming with recombinant adenoviral vectors that express Gn proteins and subsequent boosting with Gn proteins, have resulted in complete protection against SFTSV in animal models, inducing robust humoral and T cell-mediated immune responses [254]. Additionally, an innovative mRNA-based vaccine delivered via engineered Salmonella has demonstrated potent immunogenicity in mouse models. The vaccine targets multiple SFTSV antigens, elicits humoral and cellular immune responses, and provides a potential route for effective vaccination [255]. While these advances are encouraging, SFTS vaccine development is still in experimental phase, necessitating continued research and clinical trials to validate efficacy and translate research findings into viable preventive and therapeutic interventions.

## 15. Influenza Virus

Influenza virus diagnostics and therapeutics play a crucial role in maintaining global health by addressing challenges of infection induced by influenza. It is a highly mutating virus responsible for seasonal epidemics and pandemics, highlighting continuous efforts to tackle this challenge. There has been significant advancement for the early detection of influenza virus. Conventional diagnostic approaches are composed of PCR, hemagglutination assays and immunochromatography [256]. Recently, various types of biosensors have emerged as an advanced tool to detect influenza with substantial enhancement in specificity and sensitivity. Such methods are based on electrochemical, gene and impedance biosensors. These innovations have enabled significant improvements in influenza diagnostics [257]. Early diagnosis is crucial for prompt antiviral treatment. Neuraminidase and RNA polymerase inhibitors have demonstrated potential as antiviral therapies if administered at the early stage of influenza infection. However, these are facing limitations due to the emergence of resistant influenza strains [258]. Emerging therapies such as monoclonal antibodies and RNA-based treatment provide better protection against influenza [259]. Advancements in targeted drug delivery have improved efficacy and therapeutic outcomes [260]. There has been continuous improvement in influenza vaccines to tackle the seasonal flu caused by emerging strains. Based on WHO findings, quadrivalent inactivated vaccines are periodically updated to match the circulating strains [261]. Moreover, efforts are devoted to design universal vaccines for influenza for broad range protection against diverse strains [262].

A universal influenza vaccine could eliminate the need for annual vaccination and reformulation. Multiple platforms are being explored. FluMos-v2 is a self-assembled nanoparticle-based vaccine displaying hemagglutinin proteins of six influenza strains, which include 4 influenza A, and 2 influenza B strains. It is developed by NIAID and has entered the Phase 1 trial [263]. Investigational universal vaccines targeting headless HA include H1ssF and EBS-UFV-001. H1ssF is a ferritin nanoparticle-based vaccine that displays the stem region of H1HA. Findings of the Phase 1 trial have shown safety and immunogenicity against group 1 influenza viruses [264]. EBS-UFV-001 is a self-assembling nanoparticle-based vaccine displaying hemagglutinin antigens from group 1 and 2 of influenza A viruses, with an aim to provide protection against diverse and emerging influenza A strains (NCT05155319). Furthermore, a computationally optimized broadly reactive antigen (COBRA) approach has been employed for designing HA and NA antigens. Multivalent formulations induced immune responses and protected mice against a broad range of influenza subtypes. It could provide protection against multiple seasonal and pre-pandemic strains, reducing the necessity of annual reformulation and strengthening pandemic preparedness [265]. Combination vaccines have been developed for providing simultaneous protection against influenza and COVID-19. It has reflected promising results for combating multiple respiratory threats [266].

The advancement in mRNA vaccine delivery based on innovative nanotechnology platforms and non-invasive administration has improved efficacy and reduced toxicity. It has opened new avenues in the targeted delivery of mRNA vaccines for viral infections including influenza. The mRNA vaccines have benefits of rapid production, which offer the advantages of rapid response to mutations of emerging strain of influenza viruses. Furthermore, its ability to elicit T cell responses is beneficial to clear the viral infection [267,268]. Various studies have advanced to clinical trials for the mRNA-based influenza vaccine. For instance, the GSK vaccine candidate has demonstrated promising results in Phase 2 trial for influenza A and B strains (NCT06431607). Moreover, Moderna’s mRNA-1010 vaccine candidate has shown sustained humoral immunity persisting for 6 months and robust T cell responses [269]. The RNA-based vaccines for influenza have great potential to replace traditional platforms, facilitating rapid production [270]. The future of influenza vaccine development is based on advancements in universal vaccine for providing broader and effective protection against different strains. This could significantly enhance global health by addressing both seasonal infection and pandemic preparedness [271].

## 16. Challenges and Future Perspectives

**Figure 2:**
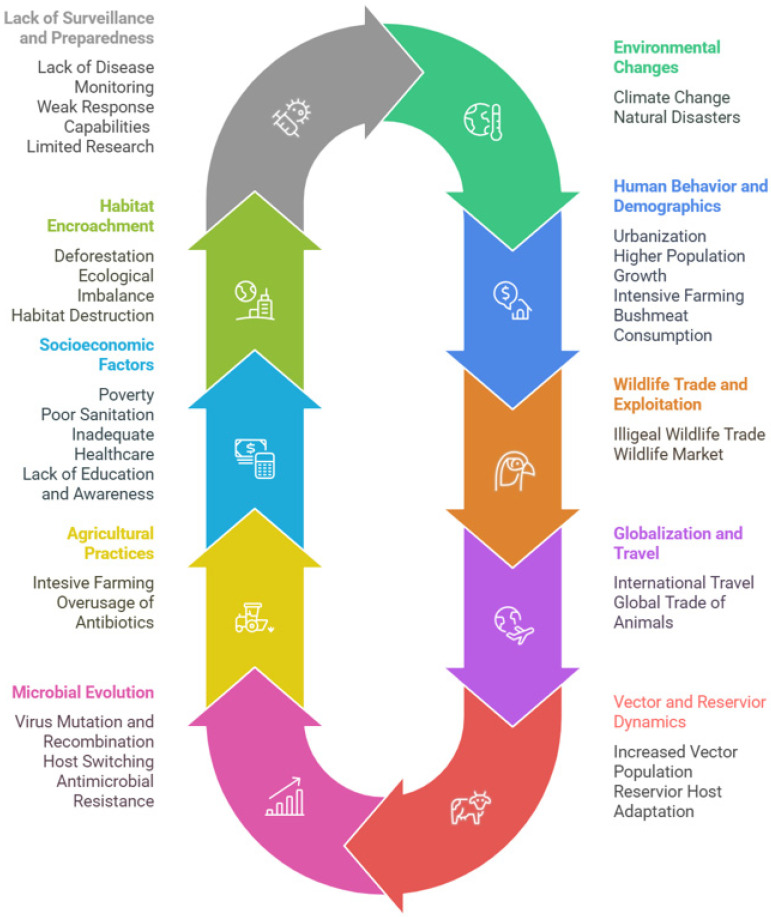
An overview of the factors driving the transmission of zoonotic viral infections.

Zoonotic infections are spread to humans via direct contact, indirect transmission, or via vectors. People who have higher chances of getting infected include farmers, veterinary personnel, and rural communities. Elderly people and individuals with compromised immunity are more vulnerable to face the health complications raised by zoonotic illness. The capability of zoonotic viruses to undergo rapid mutations and cross the species barrier enable them to attain sustain human-to-human transmission that could lead to a pandemic. The frequent mutations, along with genetic drifts and shifts make the development of vaccine and therapeutics challenging. Viruses continuously evolve to escape immune response and develop resistance to existing vaccines and therapeutics. Climate change and habitat fragmentation are contributing factors for zoonotic infections and their transmission. Other challenges associated with zoonotic infections include delayed detection, asymptomatic carriers, the lack of effective vaccines and therapeutics, and inequity in vaccine distribution. The lack of awareness and increased mobility of human beings at global scale are also important factors accelerating the transmission of zoonotic disease [272]. Key drivers for zoonotic viral infections are interconnected as highlighted in Figure 2.

Considering pathogenic potential, strict biosafety measures are essential for conducting research on the identification and surveillance of pathogenic infections to avoid exposure. This includes laboratory licensing and pathogen handling at human, animal, and agricultural facilities [273]. Respective biosafety standards should be followed by laboratory and field staff to ensure safety [274]. Additionally, biosafety training should be conducted for those working and handling pathogens. Biosafety enforcement and effective vaccination practices could play an essential role in preventing disease transmission [275]. Awareness campaigns should be emphasized in underdeveloped countries to educate communities about the health risk of interacting with wild animals. Adopting biosafety measures may reduce the risk of zoonotic disease transmission from animals to human [276].

**Figure 3:**
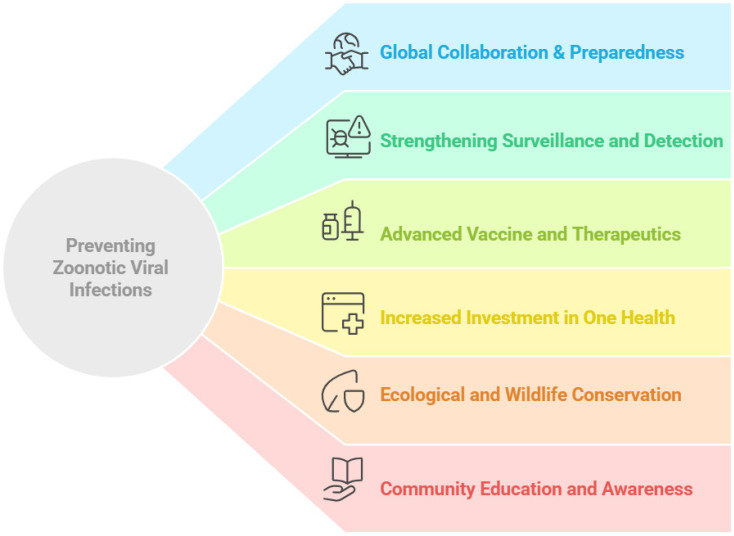
Strategies for prevention and control of zoonotic viral infections.

There has been remarkable progress in the development of mRNA vaccines, viral vector vaccines, recombinant technology, and nanoparticle-assisted targeted vaccine delivery. In clinical trial studies, several vaccine candidates have demonstrated encouraging outcomes. Early diagnostics and AI-based predictions could be further helpful to improve surveillance and prevent epidemics. Multi-dimensional efforts are needed to address the threats of zoonotic infections. A One Health approach by collaborating human, animal, and environment disciplines could play a pivotal role to combat the challenges of zoonotic infection. Besides developing innovative vaccine platforms and therapeutics, community engagement and public awareness could reduce health risks. Coordinated efforts at a global scale are essential to address the evolving problems associated with zoonotic infections. An outline of potential strategies to prevent zoonotic infections is presented in Figure 3.

## 17. Conclusions

An overview of the zoonotic infections caused by diverse viruses emphasizes early detection strategies for effective monitoring to mitigate associated health risks. Viral mutations and the emergence of new strains limit the effectiveness of existing vaccines for zoonotic infections. The emergence of drug resistance strains has reduced the outcome of antiviral therapeutics. Advancements in viral vectors and mRNA-based vaccine platforms have shown a promising response in clinical trials. A universal vaccine, particularly for influenza infection, has provided broader protection against multiple strains. Zoonotic infections continue to pose persistent problems due to host switching and adoptability. Variation in immune mechanisms across various hosts is not fully understood. Lacking licensed vaccines for different zoonotic viruses emphasizes the urgent need for intensive research to gain a deeper understanding of their pathogenesis and immune pathways. Besides innovation in vaccine development, public awareness for avoiding disease transmission could significantly reduce health risks. Global health security against zoonotic viral infections requires coordinated efforts across environmental, veterinary, and public health management.

## Data Availability

All data discussed in this article are sourced from previously published studies, which have been cited appropriately.
